# Clonal expansion of alveolar fibroblast progeny drives pulmonary fibrosis in mouse models

**DOI:** 10.1172/JCI191826

**Published:** 2025-08-28

**Authors:** Christopher Molina, Tatsuya Tsukui, Imran S. Khan, Xin Ren, Wenli Qiu, Michael Matthay, Paul Wolters, Dean Sheppard

**Affiliations:** 1 Division of Pulmonary and Critical Care Medicine, and; 2 Division of Neonatology, UCSF, San Francisco, California, USA.

**Keywords:** Cell biology, Pulmonology, Fibrosis

## Abstract

Pulmonary fibrosis (PF) has been called a fibroproliferative disease, yet the functional importance of proliferating fibroblasts to PF has not been systematically examined. In response to alveolar injury, quiescent alveolar fibroblasts differentiate into fibrotic fibroblasts that express high amounts of collagens. However, what role, if any, proliferation plays in the accumulation of fibrotic fibroblasts has remained unclear. Using 5-ethynyl-2′-deoxyuridine (EdU) incorporation, genetic lineage tracing, and single-cell RNA-Seq, we delineated the proliferation dynamics of lung fibroblasts during post-injury fibrogenesis. We found substantial DNA replication in progeny of alveolar fibroblasts in 2 independent models of PF. Lineage labeling revealed clonal expansion of these fibroblast descendants principally in regions of fibrotic remodeling. The transcriptome of proliferating fibroblasts closely resembled that of fibrotic fibroblasts, suggesting that fibroblasts can first differentiate into fibrotic fibroblasts and then proliferate. Genetic ablation of proliferating fibroblasts and selective inhibition of cytokinesis in alveolar fibroblast descendants significantly mitigated PF and rescued lung function. Furthermore, fibroblasts in precision-cut lung slices from human fibrotic lungs exhibited higher proliferation rates than did those in nondiseased lungs. Together, this work establishes fibroblast proliferation as a critical driver of PF and suggests that specifically targeting fibroblast proliferation could be a new therapeutic strategy for fibrotic diseases.

## Abstract 2

Clonal expansion of a fibroblast subpopulation drives pulmonary fibrosis, suggesting targeted inhibition as a novel therapeutic strategy.

## Introduction

Pulmonary fibrosis (PF) has been characterized as a progressive fibroproliferative disease. However, the evidence supporting an important role for fibroblast proliferation in human fibrotic lungs is controversial. Numerous studies have reported both enhanced (1–9) and diminished (10–19) proliferative capacities of fibroblasts derived from human fibrotic lungs. These discrepancies likely stem from variations in culture conditions, cell purification techniques, and heterogeneity in the distribution of lesions typically encountered in fibrotic lungs (20, 21).

Given the limited effectiveness of current therapies for IPF, it is crucial to deepen our understanding of the mechanisms underlying the progressive nature of this disease. Progressive collagen deposition in IPF is thought to originate from differentiated *CTHRC1*^+^ fibrotic fibroblasts (22, 23). These *CTHRC1*^+^ fibroblasts can assemble into pathologic aggregates called fibroblastic foci (22, 24), and increasing numbers of these foci (25, 26) and higher bulk *CTHRC1* expression correlate with more severe disease (27). These foci exhibit substantial variability in size, which could indicate temporal growth, with the smallest foci occurring at the most recent sites of injury (28) and then dynamically expanding outward, forming an invasive fibrotic front (29). However, the contribution of fibroblast proliferation to the formation of these pathologic fibroblast aggregates remains unresolved, with studies variably reporting either enriched (30–32) or diminished (29, 33–37) staining of proliferation markers in fibroblastic foci.

Currently, it is not possible to directly examine the functional importance of fibroblast proliferation in human PF. Consequently, extensive research has been directed toward understanding fibroblast proliferation in rodent models. While most of these rodent studies have suggested a role for proliferating mesenchymal cells contributing to lung fibrosis (38–50), other studies have questioned the importance of this effect (51–53). These inconsistencies could be in part explained by a limited understanding of fibroblast heterogeneity in fibrotic lungs and the absence of reliable tools to label and manipulate distinct fibroblast subsets.

As a result, no studies have definitively demonstrated that in vivo fibroblast proliferation is a critical driver of PF. One recent study utilizing Pdgfra-CreER in an influenza model reported a reduction in both lung injury severity and dysplastic epithelial regeneration when *Ect2* was deleted to inhibit cytokinesis in *Pdgfra*^+^ fibroblasts (54), but did not directly examine fibrosis as an endpoint. *Ect2*, a Rho GDP exchange factor (RhoGEF), plays an important role in both cytokinesis and cell migration (55), so the previously cited study (54) did not formally determine whether the effect on dysplastic regeneration was due to inhibition of cytokinesis or fibroblast migration. Also, since Pdgfra labels a heterogenous group of lung fibroblasts, including normal alveolar and adventitial fibroblasts (22), that study did not definitively determine which population was responsible for the effects observed.

Recent single-cell RNA (scRNA) data have identified signal peptide, CUB domain and EGF like domain containing 2 (*Scube2*) as a marker of normal alveolar fibroblasts in mice (22) and demonstrated that *Scube2*^+^ alveolar fibroblasts directly support the alveolar type II stem cell niche (23). In response to alveolar insults that lead to the development of PF, *Scube2*^+^ alveolar fibroblasts are the primary cell of origin for multiple emergent fibroblast subsets, including fibrotic, inflammatory, stress-activated, and proliferating fibroblast subsets (23). On the basis of these results, we recently developed tools to precisely label resting alveolar fibroblasts (Scube2-CreER) and induced fibrotic fibroblasts (Cthrc1-CreER).

In this study, we used these tools, together with efficient (Rosa26-lox-stop-lox TdTomato) and inefficient (Brainbow2.1/+ confetti) lineage tracing, 5-ethynyl-2′-deoxyuridine (EdU) labeling and genetic deletion of *Ect2* (which inhibits cytokinesis and migration) and *Esco2* (which impairs chromatin cohesion and thus leads to the death of proliferating cells) to directly examine the extent, functional consequence, and molecular characteristics of fibroblast proliferation in 2 distinct models of PF induced by silica or bleomycin. We also reanalyzed single-cell RNA-Seq (scRNA-Seq) data from human lungs and analyze fibroblast proliferation in human precision-cut lung slices (PCLSs) to assess the relevance of murine findings to PF in humans. We observed substantial fibroblast proliferation in both murine models and show that the predominant population of proliferating fibroblasts were *Cthrc1*^+^ fibrotic fibroblasts derived from *Scube2*^+^ lineage–traced alveolar fibroblasts. These descendants of alveolar fibroblasts clonally expanded, forming pathologic fibroblast clusters in regions of fibrotic remodeling. We show that selective inhibition of cytokinesis or migration (by deletion of *Ect2*) or induction of cell death in proliferating cells (by deleting *Esco2*) in cells derived from alveolar fibroblasts diminished the formation of pathologic fibroblast clusters, reduced collagen accumulation, and rescued organ function in 2 in vivo models of PF. Furthermore, we show that human lung fibroblasts in fibrotic lung slices proliferated more extensively than did fibroblasts in normal human lung slices and that in scRNA-Seq data from human lungs, *CTHRC1*^+^ fibroblasts had the highest rate of expression of genes associated with cell-cycle progression. These findings strongly suggest that, in response to fibrotic alveolar injury, alveolar fibroblasts can differentiate into fibrotic fibroblasts and then proliferate. This proliferation significantly contributed to the formation of fibroblast aggregates, the accumulation of excess collagen and the subsequent impairment of gas exchange and lung function. Since fibroblast proliferation also appeared to occur in fibrotic fibroblasts in human fibrotic lungs, these results suggest that targeted inhibition of fibroblast proliferation could be a viable strategy for the treatment of PF.

## Results

### Evaluating lung fibroblast proliferation dynamics in 2 in vivo models of PF.

Given the pivotal role of fibroblasts in lung scarring, we postulated that fibroblast proliferation is a fundamental mechanism in fibrotic lung remodeling. To this end, we analyzed the proliferation dynamics of lineage-negative (CD45^–^, CD31^–^, EPCAM^–^, MCAM^–^) lung fibroblasts in 2 in vivo models of lung fibrosis. We used adult C56BL/6 WT mice, challenging them with a single intra-airway dose of either bleomycin or silica, followed by daily injections of EdU for 4 days before harvesting at intervals from post-injury days 0–16. This allowed us to quantify the rolling average of proliferating fibroblasts at these time points (Supplemental Figure 1, A and B; supplemental material available online with this article; https://doi.org/10.1172/JCI191826DS1). Our findings indicate significant rates of EdU incorporation in both models, with the peak effect occurring between post-injury days 4 and 8 in silica-treated lungs and days 8–12 in bleomycin-treated lungs (Supplemental Figure 1C). In both models a substantial fraction of fibroblasts had incorporated EdU during all time intervals studied.

### Origin and fate of proliferating fibroblasts in lung fibrosis models.

**Figure 1:**
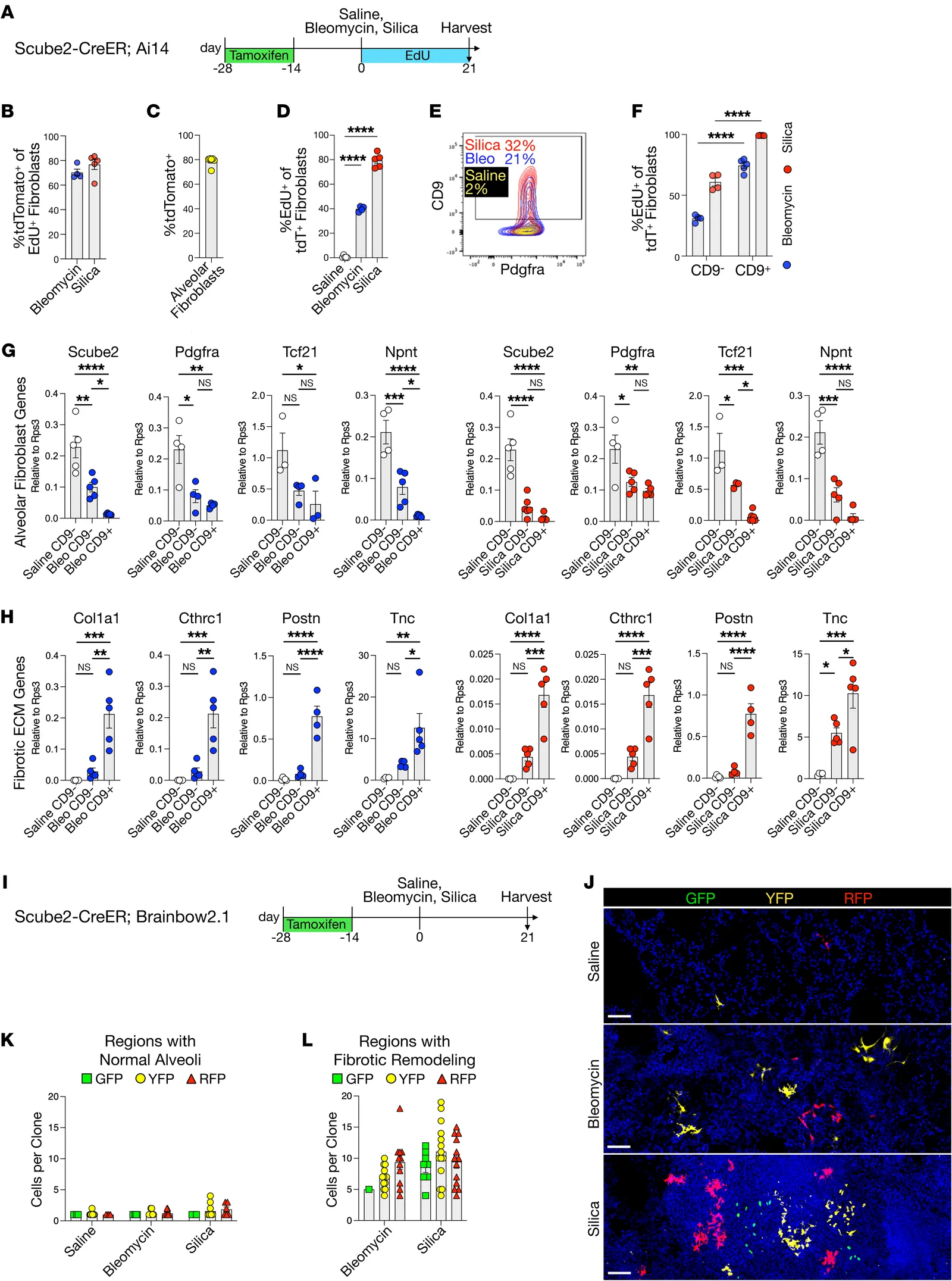
Alveolar fibroblast descendants proliferate in regions of fibrotic remodeling and adopt a profibrotic phenotype. (**A**) Alveolar fibroblasts in Scube2-CreER/Rosa26-Ai14 mice were labeled with tdTomato via tamoxifen injection, subjected to bleomycin or silica challenge, and then the mice were treated with EdU in the drinking water to label proliferating cells. On day 21, tdTomato^+^ fibroblasts were quantified and sorted via FACS for qPCR analysis. (**B**) In bleomycin-treated lungs, 70.3% (± 5.4%) of all lineage-negative fibroblasts that proliferated at least once were tdTomato^+^, compared with 76.8% (± 9.0%) in silica-treated lungs. (**C**) In saline-control mice, the labeling efficiency of alveolar fibroblasts by Scube2-CreER was 78.1% (± 3.9%). (**D**) Analysis revealed that 39.9% (± 2.2%) of tdTomato^+^ fibroblasts proliferated at least once in bleomycin-treated lungs compared with 79.4% (± 5.7%) in silica-treated lungs and 1.3% (± 0.9%) in saline-control lungs. (**E**) CD9 upregulation was noted in 20%–30% of tdTomato^+^ fibroblasts under fibrotic conditions. (**F**) CD9^+^tdTomato^+^ fibroblasts showed higher proliferation rates compared with CD9^–^tdTomato^+^ fibroblasts in bleomycin- and silica-treated lungs. (**G** and **H**) qPCR of sorted cells showed CD9^+^ fibroblasts, preferentially downregulated markers of quiescent alveolar fibroblasts (**G**), and upregulated fibrotic ECM genes (**H**). (**I**) Sparse labeling with GFP, YFP, or RFP in Scube2-CreER Rosa26-Brainbow2.1/+ mice enabled tracking of fibroblast clonal expansion. (**J**–**L**) Confocal imaging of cleared lung sections from treated mice revealed clonal expansions in fibrotic areas, quantified by cellular density per clone in fibrotic versus normal regions. Scale bars: 50 μm. *P < 0.05, **P < 0.01, ***P < 0.001, and ****P < 0.0001, by 2-way ANOVA (**D** and **F**) and 1-way ANOVA (**G** and **H**). Data represent the mean ± SEM.

Recent studies demonstrated a progressive increase in a population of collagen triple helix repeat containing 1 (*Cthrc1*^+^) fibrotic fibroblasts in response to bleomycin and showed that *Scube2*^+^ alveolar fibroblasts are the major progenitor source of these cells (23). However, whether aggregates of fibrotic fibroblasts simply emerge from existing alveolar fibroblasts or expand as a result of proliferation remains unclear. To begin to delineate the role of proliferation, we utilized Scube2-CreER Rosa26-Ai14 mice to label alveolar fibroblasts with tdTomato, induced fibrosis using bleomycin or silica, and administered EdU in the drinking water over the first 21 days after injury (Figure 1A). Remarkably, an average of 73.9% (± 8.0%) of EdU-labeled fibroblasts were labeled with tdTomato (Figure 1B), closely mirroring the 78.1% labeling efficiency of alveolar fibroblasts by Scube2-CreER in saline controls (Figure 1C) (22), suggesting that the vast majority of fibroblasts incorporating EdU in each fibrosis model were derived from alveolar fibroblasts.

We observed that 39.9% of tdTomato-labeled fibroblasts in bleomycin-injured lungs and 79.4% in silica-injured lungs were EdU labeled (Figure 1D). We previously showed that the cell-surface marker CD9 is not normally expressed by alveolar fibroblasts, but at 21 days after bleomycin treatment, CD9 expression is specifically induced in fibrotic fibroblasts (22). We therefore sorted tdTomato^+^ cells for CD9 expression at the 21-day endpoint of EdU labeling and compared EdU incorporation in CD9^+^ cells with CD9^–^ cells. In both models, we observed that a higher fraction of CD9^+^ cells had undergone at least 1 round of DNA replication (as detected by EdU labeling), with values of 74.5% after bleomycin and 99.2% after silica (Figure 1, E and F). To define the fate of proliferating fibroblasts, we performed quantitative PCR (qPCR) on sorted cells harvested at post-treatment day 21. CD9^+^tdTomato^+^ fibroblasts showed marked downregulation of alveolar fibroblast markers (*Scube2*, *Pdgfra*, *Tcf21*, *Npnt*) and upregulation of fibrotic matrix genes and markers characteristic of fibrotic fibroblasts (*Col1a1*, *Cthrc1*, *Postn*, *Tnc*), consistent with a transcriptional shift from an alveolar to a fibrotic fibroblast phenotype (Figure 1, G and H).

These results suggest that proliferating fibroblasts predominantly arose from alveolar fibroblast progenitors and that most CD9^+^ fibrotic fibroblasts emerged from cells that had undergone at least 1 round of DNA replication.

### Spatial distribution of fibroblast proliferation in fibrotic lungs.

To further elucidate the spatial distribution and extent of fibroblast proliferation in fibrotic lungs, we used Scube2-CreER Brainbow2.1/+ confetti mice. After administering tamoxifen to induce sparse labeling of alveolar fibroblasts and their descendants with GFP, YFP, or RFP, we subjected the mice to challenges with bleomycin, silica, or saline (Figure 1I). Confocal imaging of cleared thick sections demonstrated clear-cut clonal expansion of alveolar fibroblast progeny, and this expansion predominantly occurred in areas of dense fibrotic remodeling (Figure 1, J–L). These results support the conclusion that EdU incorporation in alveolar fibroblast progeny is a true marker of cell proliferation and identify regions of dense fibrotic remodeling as the principal site of clonal expansion in both fibrosis models.

### Heterogeneity of proliferating fibroblasts in lung fibrosis.

**Figure 2:**
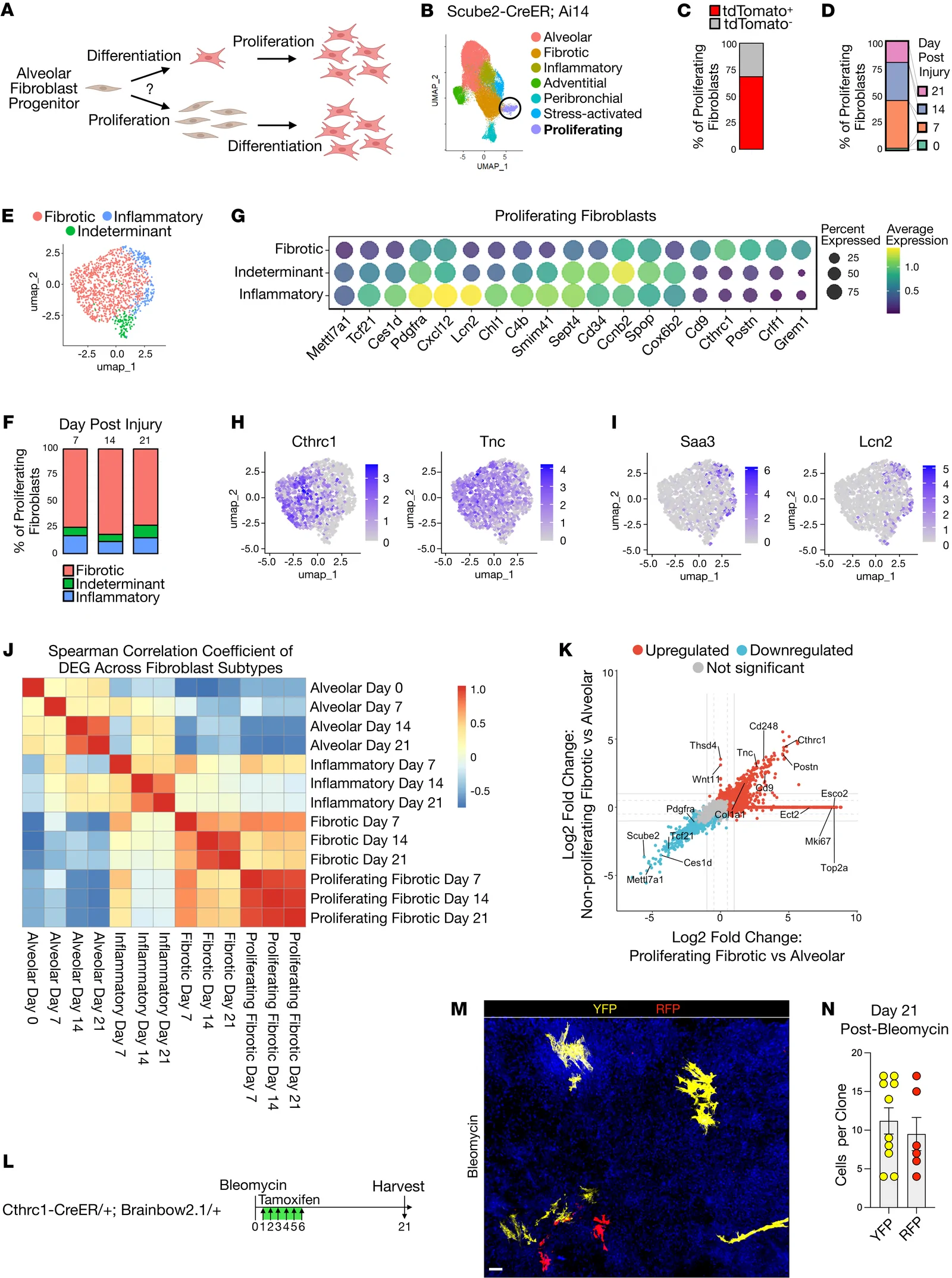
Cthrc1^+^ fibrotic fibroblasts are the dominant proliferating fibroblast subtype in fibrotic lungs. (**A**) Potential pathways of fibroblast proliferation and differentiation in fibrotic lungs (**B**) UMAP visualization from Tsukui et al. (23), showing fibroblasts harvested from Scube2-CreER R26-Ai14 mice at days 0, 7, 14, and 21 after bleomycin injury. The proliferating fibroblast cluster was subsetted and reclustered for downstream analysis. Figure 2A was partially created in BioRender (https://BioRender.com/b92n121). (**C**) Most proliferating fibroblasts were lineage labeled by tdTomato. (**D**) Maximal fibroblast proliferation was observed at day 7 after bleomycin injury. (**E**) Reclustering identified 3 distinct proliferating fibroblast subpopulations: fibrotic, inflammatory, and indeterminate fibroblasts. (**F**) Fibrotic fibroblasts consistently emerged as the predominant proliferating type across all time points. (**G**) Bubble plot and feature plots (**H** and **I**) displaying marker gene expression profiles across subtypes. (**J** and **K**) Spearman correlation and scatter plot analyses confirmed the transcriptomic similarities between proliferating and nonproliferating fibrotic fibroblasts. Dashed gray line ± 0.5 average log_2_ FC; solid gray line ± 1 average log_2_ FC. (**L**–**N**) Longitudinal tracking of *Cthrc1*^+^ fibrotic fibroblast clonal expansion in bleomycin-treated lungs, visualized by confocal microscopy using cleared thick sections, which showed substantial cellular proliferation within fibrotic regions. Cthrc1-CreER labeled a small percentage of lineage^+^ cells at baseline, so only cytoplasmic YFP^+^ and cytoplasmic RFP^+^ clones with clear fibroblast morphology were quantified. Scale bar: 30 μm.

Generally, it has been thought that fibroblasts proliferate before they differentiate into extracellular matrix–producing (ECM-producing) fibroblasts (56–59). To explore the molecular phenotype of proliferating fibroblasts, we leveraged our previously published scRNA-Seq dataset (23), which highlighted the emergence of distinct fibroblast subpopulations in bleomycin-induced fibrotic lungs — fibrotic, inflammatory, and proliferative fibroblasts (Figure 2B). Our reanalysis of the proliferative fibroblast cluster (Figure 2C) confirmed that most of the proliferating fibroblasts originated from Scube2-CreER alveolar fibroblasts, aligning with our continuous EdU-labeling results (Figure 1B). Notably, the largest fraction of proliferating fibroblasts were present at day 7 after injury (Figure 2D), which we verified using 24-hour EdU pulse injections in Scube2-CreER Ai14 mice (Supplemental Figure 2).

Further reclustering of the proliferating fibroblast population revealed 3 transcriptionally distinct subtypes: fibrotic, inflammatory, and a smaller, indeterminate group (Figure 2, E–I). The inflammatory subtype was characterized by the expression of inflammatory genes such as *Saa3*, *Lcn2*, *Cxcl12*, and *C4b* (Figure 2, G and I), which overlap with markers of the “transition” fibroblasts described by Mayr, Sengupta, and co-authors and Konkimalla et al. (Supplemental Figure 3) (60, 61). The proliferating fibrotic subtype predominantly expressed genes that are typically upregulated in fibrotic fibroblasts, such as *Cthrc1*, *Tnc*, *Postn*, and *Grem1* (Figure 2, G and H) (23, 24, 27). Surprisingly, the Cthrc1^+^ fibrotic fibroblast was the dominant proliferative cell subpopulation across all time points analyzed (Figure 2F).

To delineate how proliferative fibrotic fibroblasts compare with nonproliferative fibrotic fibroblasts at the global transcriptome level, we performed Spearman’s correlation analysis across fibroblast subtypes, which confirmed that proliferative fibrotic fibroblasts closely resemble nonproliferative fibrotic fibroblasts (Figure 2J). A comparison of the differential gene expression profiles between these 2 states showed nearly identical patterns in their downregulation of alveolar fibroblast genes such as *Scube2*, *Pdgfra*, *Tcf21*, and *Mettl7a1* and upregulation of CD9 and fibrotic ECM genes such as *Col1a1*, *Cthrc1*, *Postn*, and *Tnc* (Figure 2K). Unique markers distinguishing the proliferative from nonproliferative fibrotic fibroblasts predominantly included cell-cycle genes like *Mki67*, *Top2a*, *Esco2*, and *Ect2* (Figure 2K).

To further test the ability of differentiated fibrotic fibroblasts to proliferate, we treated Cthrc1-CreER Brainbow2.1/+ mice with bleomycin, and then administered tamoxifen on days 1–6 to sparsely label emerging fibrotic fibroblasts with either red fluorescent protein (RFP) or yellow fluorescent protein (YFP) (Figure 2L). By post-injury day 21, clonal expansion of Cthrc1-CreER–labeled fibroblasts was evident (Figure 2, M and N), mirroring patterns observed in bleomycin-treated Scube2-CreER Brainbow2.1/+ mice (Figure 1L) and confirming that alveolar progeny that had already differentiated into fibrotic fibroblasts could undergo substantial clonal expansion (Figure 2A).

### Functional role of proliferating fibroblasts in fibrotic lung injury models.

**Figure 3:**
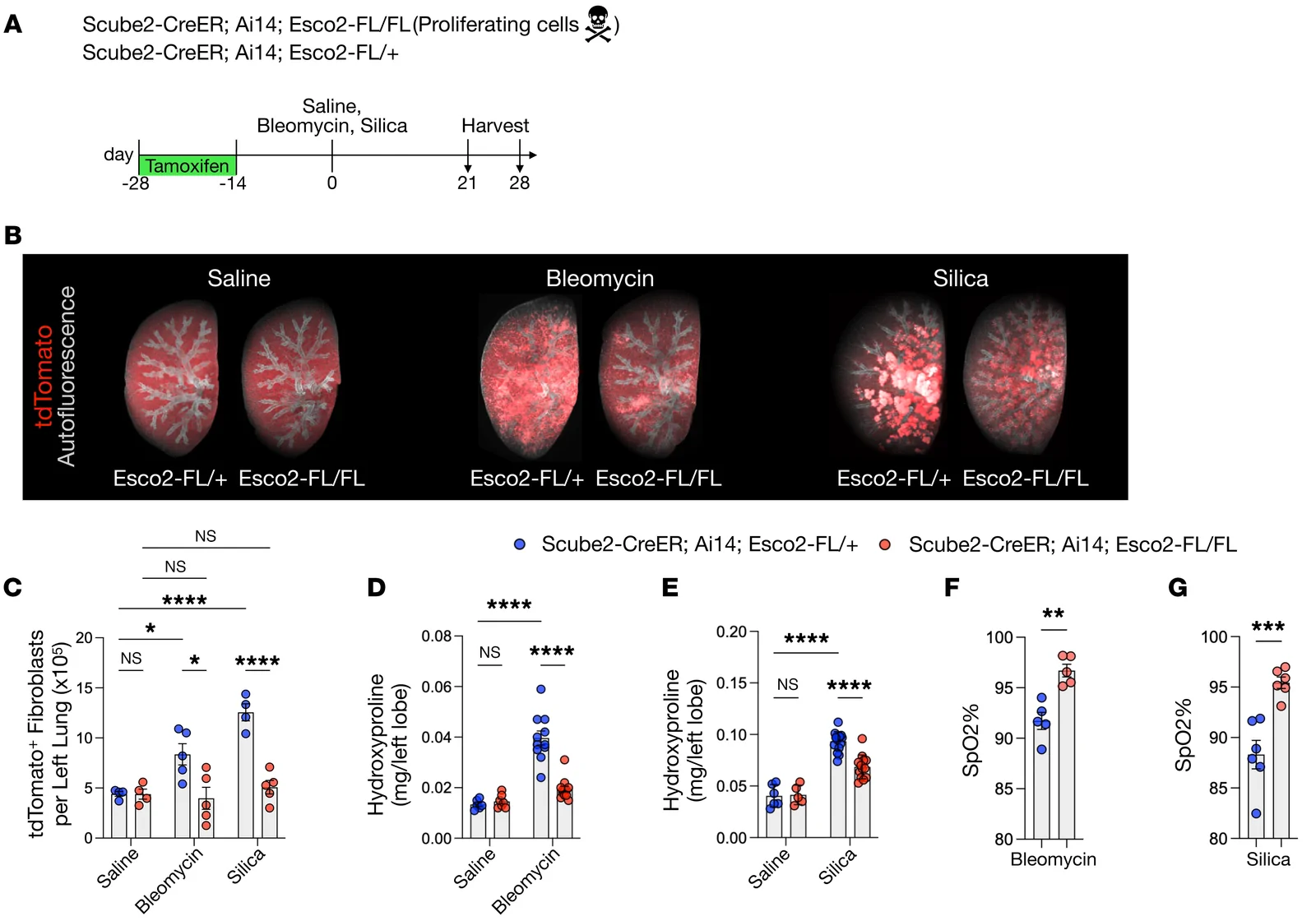
Genetic deletion of proliferating fibroblasts reduces pulmonary fibrosis and restores lung function. (**A**) Tamoxifen was administered to Scube2-CreER Ai14 Esco2-FL/FL mice to both label alveolar fibroblasts with tdTomato and delete *Esco2*, selectively targeting proliferating progeny for ablation. Lungs were harvested on day 21 for imaging and flow cytometry, and on day 28 for hydroxyproline quantification. (**B**) Maximum intensity projections of cleared right upper lobes, imaged by light-sheet microscopy, which highlighted tdTomato^+^ cells (red) against autofluorescent airways (gray). (**C**) FACS quantification showed that *Esco2* deletion reduced tdTomato^+^ fibroblasts by 53% in bleomycin-treated lungs and by 60% in silica-treated lungs (*n* = 8 saline, *n* = 11 bleomycin, *n* = 9 silica). (**D** and **E**) Hydroxyproline assays demonstrated reduced lung fibrosis in both bleomycin- and silica-treated Esco2-FL/FL lungs (*n* = 22 saline, *n* = 23 bleomycin, *n* = 29 silica). (**F** and **G**) Improved lung oxygenation was quantified via pulse oximetry in bleomycin- and silica-treated Esco2-FL/FL mice (*n* = 10 bleomycin, *n* = 12 silica). *P < 0.05, **P < 0.01, ***P < 0.001, and ****P < 0.0001, by 2-way ANOVA with Tukey’s multiple-comparison test (**C**–**E**) and unpaired parametric 2-tailed *t* tests (**F** and **G**). Data represent the mean ± SEM.

To assess the effects of eliminating proliferating fibroblasts, we treated Scube2-CreER Ai14 Esco2-FL/FL mice with tamoxifen, which activated tdTomato labeling and deleted the *Esco2* gene in alveolar fibroblasts and their descendants (Supplemental Figure 4B). *Esco2* is a crucial element of the chromatin cohesion complex, and loss of *Esco2* in proliferating cells results in cell apoptosis (62). Thus, deletion of *Esco2* allowed targeting of proliferating fibroblasts for apoptosis during lung fibrogenesis induced by bleomycin or silica (Figure 3A). At day 21, whole lung imaging revealed marked reductions in dense tdTomato^+^ fibroblast aggregates in *Esco2*-deficient mice compared with controls in both the bleomycin and silica models (Figure 3B and Supplemental Figure 4A). Flow cytometry quantification confirmed a decrease in total tdTomato^+^ fibroblasts in the treated Scube2-CreER Ai14; Esco2-FL/FL mice (Figure 3C). Consistent with this reduction, Esco2-FL/FL mice also showed decreased fibrosis and improved lung oxygenation, as assessed by hydroxyproline, Picrosirius red, and pulse oximetry measurements (Figure 3, D–G, and Supplemental Figure 4C).

**Figure 4:**
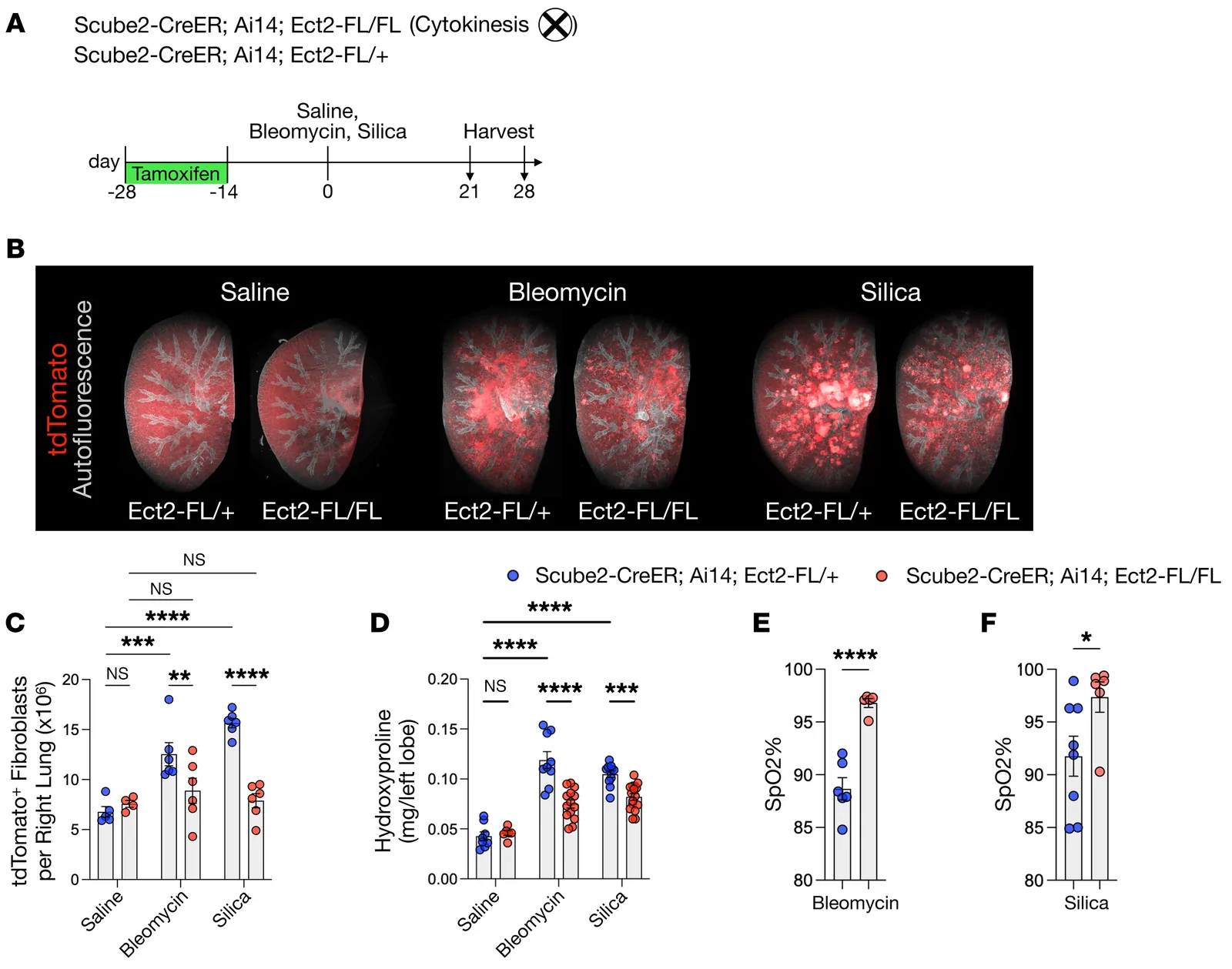
Genetic inhibition of fibroblast proliferation ameliorates lung fibrosis and restores lung function. (**A**) Tamoxifen treatment of Scube2-CreER Ai14 Ect2-FL/FL mice induced tdTomato labeling and *Ect2* deletion specifically in alveolar fibroblasts, resulting in selective inhibition of cytokinesis in their proliferating progeny. Lungs were harvested on day 21 for imaging and flow cytometry and on day 28 for hydroxyproline quantification. (**B**) Maximum intensity projections of cleared right upper lobe sections visualized via light-sheet microscopy show tdTomato^+^ cells (red) and autofluorescent airways (gray). (**C**) FACS quantification revealed that *Ect2* deletion reduced tdTomato^+^ fibroblasts by 29% in bleomycin-treated lungs and by 50% in silica-treated lungs (*n* = 9 saline, *n* = 12 bleomycin, *n* = 12 silica). (**D**) Hydroxyproline assays indicated decreased fibrosis in Ect2-FL/FL mice challenged with bleomycin or silica (*n* = 14 saline, *n* = 23 bleomycin, *n* = 26 silica). (**E** and **F**) Pulse oximetry measurements showed enhanced lung oxygenation in Ect2-FL/FL mice treated with bleomycin or silica (*n* = 10 bleomycin, *n* = 14 silica). *P < 0.05, **P < 0.01, ***P < 0.001, and ****P < 0.0001, by 2-way ANOVA with Tukey’s correction for multiple comparisons (**C** and **D**) and unpaired parametric 2-tailed *t* tests (**E** and **F**). Data represent the mean ± SEM.

To explore the consequences of selective inhibition of fibroblast proliferation without killing, we deleted *Ect2* in Scube2-CreER Ai14 Ect2-FL/FL mice to impair cytokinesis (55) in tdTomato^+^ alveolar fibroblasts under similar conditions (Figure 4A and Supplemental Figure 5B). At day post-injury day 21, both confocal imaging of thin sections and whole-lung light sheet microscopy revealed marked reductions in tdTomato^+^ fibroblast aggregates in *Ect2*-deficient mice (Figure 4B and Supplemental Figure 5A). This was corroborated by flow cytometric data showing a reduction in the total number of tdTomato^+^ fibroblasts (Figure 4C), and in hydroxyproline and Picrosirius red assays confirming decreased fibrosis in these mice (Figure 4D and Supplemental Figure 5C). Pulse oximetry revealed significant improvements in oxygenation in the *Ect2* model, emphasizing the beneficial effects of inhibiting fibroblast proliferation on overall lung health (Figure 4, E and F). Together, these loss-of-function experiments demonstrate that fibroblast proliferation worsened fibrosis severity and lung function in 2 in vivo models of PF.

### Diversity and dynamics of proliferating fibroblasts in human fibrotic lungs.

**Figure 5:**
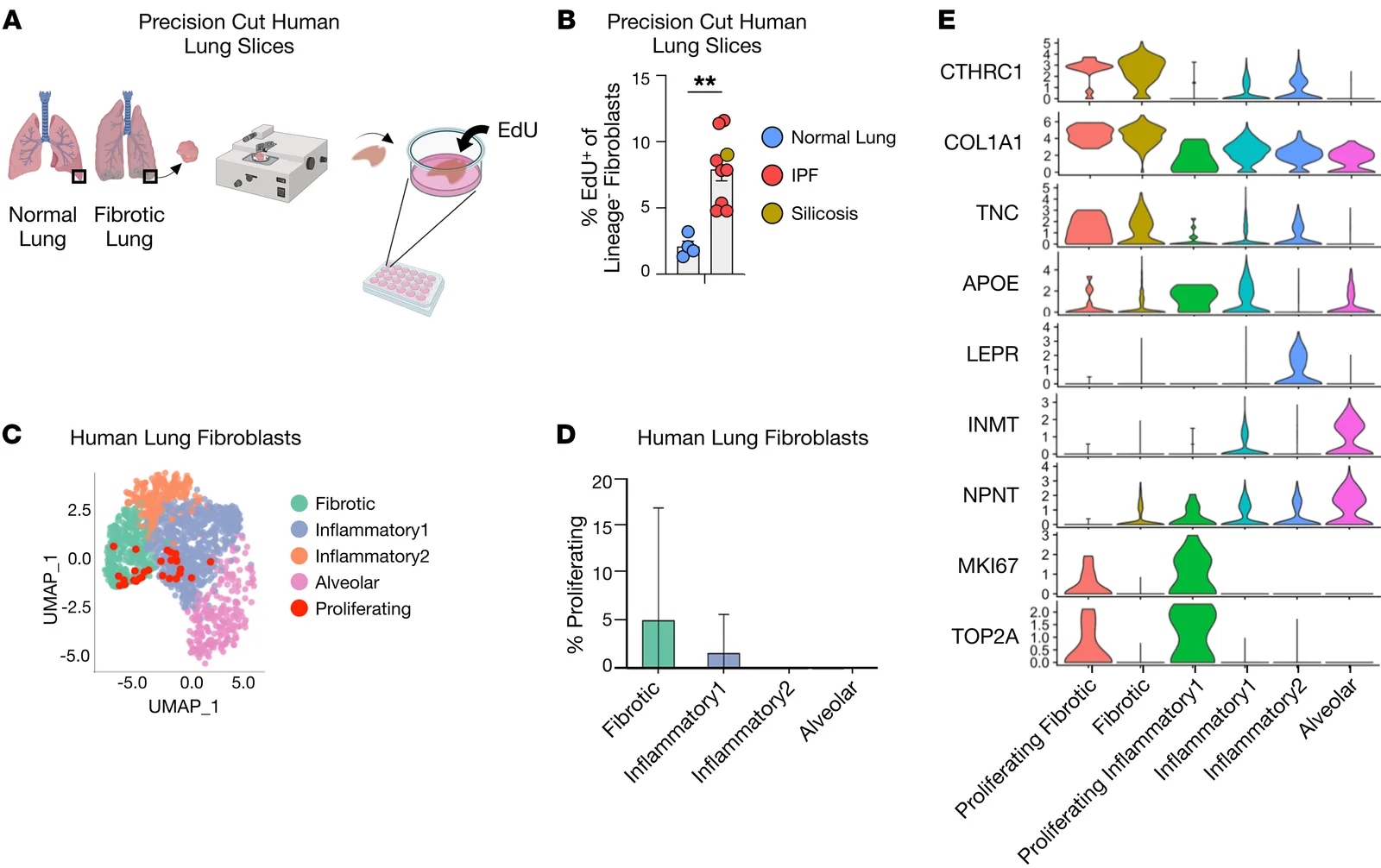
Heterogeneity of proliferating fibroblasts in human fibrotic lungs. (**A**) Human PCLSs from both fibrotic and nondiseased donors were cultured in 1% FBS DMEM supplemented with EdU for 2 days and then (**B**) analyzed by flow cytometry (fibrotic lung donors, *n* = 9; nondiseased lung donors, *n* = 4). **P < 0.01, by unpaired parametric 2-tailed *t* test. Data represent the mean ± SEM. (**C**) Reanalysis of the Habermann et al. (63) Idiopathic Pulmonary Fibrosis Cell Atlas by Tsukui et al. (23) illustrating the presence of alveolar fibroblasts and 3 distinct pathologic fibroblast subtypes in human fibrotic lungs: fibrotic, inflammatory-1, and inflammatory-2. Cell-cycle scoring revealed proliferating fibroblasts predominantly within the fibrotic and inflammatory-1 fibroblast subpopulations. (**D**) Proportion of proliferating cells within each fibroblast subtype. (**E**) Violin plots delineating expression profiles of marker genes across fibroblast subtypes.

We hypothesized that the proliferative responses of fibroblasts in murine fibrotic lungs may exhibit some similarities to those in human fibrotic lungs. Thus, we conducted EdU labeling and FACS quantification of proliferation rates in lineage-negative (CD45^–^, CD31^–^, EPCAM^–^, MCAM^–^) human lung fibroblasts derived from PCLSs of age-matched, nondiseased donors and fibrotic lung explants (Figure 5A and Supplemental Table 2). These data show that human lung fibroblasts in fibrotic lung PCLSs had a similarly enhanced proliferative capacity (Figure 5B).

Building on our findings in murine models, we hypothesized that these proliferating fibroblasts in human fibrotic lungs could similarly adopt a profibrotic phenotype. To begin testing this, we performed ISH on IPF PCLSs and identified EdU^+^CTHRC1^+^ cells, indicating that cells undergoing DNA replication in human lung tissue can acquire a profibrotic fate (Supplemental Figure 6A). Next, we analyzed the transcriptomic diversity of proliferating fibroblasts using the Human Idiopathic Pulmonary Fibrosis Single-Cell Atlas by Habermann et al., reanalyzed by Tsukui et al., which revealed the emergence of 3 distinct pathologic fibroblast subpopulations in fibrotic lungs (Figure 5C) (23, 63). To identify proliferating fibroblasts, we performed cell-cycle scoring within Seurat, utilizing DBSCAN clustering to establish optimal cutoffs for cells expressing high levels of G_2_M and S-phase cell-cycle genes (Supplemental Figure 6, B–D). Fibroblasts exhibiting elevated G_2_M or S scores were re-annotated as “proliferating” and subsequently overlaid on uniform manifold approximation and projection (UMAP) plots of all alveolar and fibrosis-associated fibroblasts. This visualization demonstrated that all proliferating cells were contained within the fibrotic or inflammatory-1 subtypes (Figure 5C). Quantitative analysis confirmed that fibrotic fibroblasts trended toward higher proliferation rates compared with their inflammatory-1 counterparts (Figure 5D), consistent with observations in our bleomycin-induced murine model (Figure 2F). The expression profiles of key pathologic ECM) genes, such as *COL1A1*, *CTHRC1*, and *TNC*, were notably elevated in both proliferating and nonproliferating fibrotic fibroblasts (Figure 5E), paralleling the gene expression trends noted in murine models (Figure 2K).

## Discussion

In the current study, we demonstrate ongoing DNA replication (measured by EdU labeling) in 2 different murine models of PF. Using mice we recently described in which Scube2-CreER specifically induces recombination in alveolar fibroblasts, we show that a large fraction of the progeny of alveolar fibroblasts in both models had undergone at least 1 round of DNA replication during the first 21 days after initiation of the models and that DNA replication was enriched in progeny of alveolar fibroblasts that had differentiated into fibrotic fibroblasts, as identified by induction of the cell-surface protein CD9 and confirmed by qPCR of sorted cells. We further demonstrate that this increase in DNA replication was associated with true cell proliferation, indicated by an increase in the total number of alveolar fibroblast progeny, and with substantial clonal expansion, measured by counting colony numbers after inefficient labeling in the Brainbow mice. Furthermore, we demonstrate that clonal expansion was largely restricted to regions of dense fibrotic remodeling.

By reanalysis of our own publicly available scRNA-Seq data, we found that the overwhelming majority of fibroblasts we previously characterized as actively proliferating demonstrated patterns of gene expression that otherwise overlapped with the molecular phenotype of fibrotic fibroblasts, suggesting that the induction of proliferation may occur simultaneously with, or following, the induction of the fibrotic molecular phenotype (23). This interpretation is further supported by our observation that fibrotic fibroblasts labeled early after treatment with bleomycin using the Cthrc1-CreER line underwent a degree of clonal expansion similar to that we observed with labeling of normal alveolar fibroblasts.

Finally, we report that deletion of *Esco2* to kill proliferating alveolar fibroblast progeny or deletion of *Ect2* to inhibit cytokinesis in the same cells both caused a reduction in fibroblast expansion and led to protection from PF and the resultant hypoxemia caused by either bleomycin or silica, suggesting that fibroblast proliferation is functionally important for driving fibrosis. Our findings that proliferating fibroblasts were present even in end-stage fibrotic human lungs, that these cells often had a fibrotic molecular phenotype, and that fibroblasts in PCLSs from fibrotic human lungs were more proliferative than fibroblasts in PCLSs from normal human lungs could have clinical relevance.

Prior studies investigating the extent of fibroblast proliferation in PF models have yielded conflicting results, with some studies suggesting important contributions of mesenchymal cell proliferation to fibrosis development (38–50), whereas others have cast doubt on this conclusion (51–53). However, a major consideration in studies of mesenchymal cell proliferation involves the challenge of cellular heterogeneity within the fibroblast population (64), with uncertainty as to whether increased cell numbers reflect a generalized proliferative response or selective expansion of a subpopulation (65).

Mayr, Sengupta, and colleagues reported proliferating transitional fibroblasts and proliferating myofibroblasts similar to the *Saa3*^+^ inflammatory fibroblasts and *Cthrc1*^+^ fibrotic fibroblasts we describe here (Supplemental Figure 3) (60). Valenzi et al. showed proliferating *Cthrc1*^+^ fibrotic fibroblasts in fibrotic lungs of patients with systemic sclerosis similar to our findings in IPF (66). While it has generally been thought that fibroblasts proliferate before they differentiate into ECM-producing fibroblasts (20, 56–59), our lineage-tracing data show that lung fibroblasts can differentiate into *Cthrc1^+^* fibrotic fibroblasts and then proliferate, and raise the possibility that this trajectory could even be the most common course. Moreover, the discovery of proliferating *Cthrc1*^+^ fibrotic fibroblasts in other datasets of fibrosis in the skin, colon, liver, and heart (66–68) suggests that the therapeutic potential of selective targeting of proliferating fibroblasts may be broadly applicable to other fibrotic diseases.

We also show, for the first time to our knowledge, that proliferating fibroblasts were functionally important in driving lung fibrosis and impairing gas exchange. We used 2 orthogonal strategies, one involving specific killing of proliferating progeny of alveolar fibroblasts by deleting *Esco2* and inhibiting proliferation without inducing cell death and the second involving interference with cytokinesis by deleting *Ect2*. Each of these strategies has its own weaknesses. Deletion of *Esco2* would induce fibroblast apoptosis, which could itself induce secondary responses in nearby cells, thereby confounding the conclusion that the protective effects of this intervention were simply due to loss of proliferating fibroblasts. *Ect2* is a Rho guanidine exchange factor that inhibits cytokinesis by blocking the critical role of Rho activation in this process. However, Rho is also an important regulator of cell migration, so it is not possible to explicitly exclude inhibition of fibroblast migration as an explanation for the functional benefit of *Ect2* deletion. However, our findings that both strategies led to similar protection from PF and hypoxemia in 2 distinct injury models provide strong support for the conclusion that the proliferation of alveolar fibroblast progeny plays an important role in driving fibrotic remodeling and impairing lung function.

The differential magnitude of protection observed between our 2 models likely reflects a key mechanistic difference: in the *Ect2* model, fibroblasts failed cytokinesis but persisted as binucleate cells, presumably retaining their ability to produce collagen. In contrast, *Esco2* deletion eliminated proliferating fibroblasts, resulting in a more substantial reduction in collagen-producing cells (Figure 3C and Figure 4C). There are 2 likely explanations for the lack of an even greater reduction in tdTomato^+^ fibroblasts in Esco2-deficient lungs: (a) the deletion efficiency of *Esco2* using Scube2-CreER reached only 70%, and (b) prior studies describing the *Esco2*-deletion mouse model reported embryonic lethality between the 2- and 8-cell stages (63), suggesting that *Esco2*-deficient fibroblasts may still undergo 1–3 rounds of replication prior to undergoing apoptosis.

These findings are conceptually aligned with prior studies of cardiac and hepatic fibrosis, in which inhibition of fibroblast proliferation similarly conferred protection and improved tissue function (69–71), further reinforcing the emerging view that targeting fibroblast proliferation represents a broadly applicable strategy to limit pathologic matrix remodeling across organ systems.

Despite these advances, several important questions remain unanswered by the work described here. These include identification of the molecular drivers of fibroblast proliferation, whether these drivers are shared among models of PF, and whether similar pathways drive fibrosis in mice and humans. The similarities and differences between the signals that drive proliferation and those that drive the fibrotic phenotype also remain to be elucidated. All these questions should be the focus of future research.

It is obviously challenging to extrapolate our findings from these 2 murine models to conclusions about the functional contribution of fibroblast proliferation to human PF. However, we were encouraged to see that we could identify proliferating fibroblasts even in explants from patients with end-stage lung disease and that these proliferating fibroblasts were enriched among fibrotic fibroblasts, similar to what we observed in our murine models. A key limitation of our PCLS studies is that only 1 of our PCLS samples came from a patient with pulmonary silicosis, reflecting the scarcity of viable human silicosis tissue and underscoring the need for further studies to dissect the mechanisms that drive fibroblast expansion in this form of lung fibrosis. Nevertheless, our observation that fibroblasts in PCLSs from fibrotic human lungs were more likely to proliferate than fibroblasts in PCLSs from normal human lungs provides further support for the notion that fibroblast proliferation could be important in human PF. Testing this hypothesis directly will require the development of therapeutic interventions that specifically target proliferating fibroblasts in vivo, ideally without targeting proliferation in other cell types. Given the intractable nature and high mortality rate for patients with PF, we think our results should at least encourage efforts to develop such strategies.

## Methods

### Sex as a biological variable.

Our study examined male and female animals, and similar findings are reported for both sexes.

### Mice and bleomycin/silica treatment.

The following mouse strains were used for experiments: C56BL/6 WT mice (Jax strain no. 000664, The Jackson Laboratory); Scube2-CreER mice (generated in the Sheppard Laboratory) (23); Cthrc1-CreER mice (generated in the Sheppard Laboratory) (23); R26-Ai14 mice (Jax strain no. 007914); Brainbow2.1/Confetti mice (Jax strain no. 017492) (72); Esco2-floxed mice (Jax strain no. 030199); and Ect2-floxed mice (a gift from Alan Fields, Mayo Clinic, Jacksonville, Florida, USA) (55). Sex-matched littermates, aged 12–16 weeks, were used for the experiments. Because male mice develop more severe fibrosis after bleomycin treatment (73), males were treated with 2.5 units/kg bleomycin, and female mice were treated with 3 units/kg bleomycin in 75 μL saline by oropharyngeal aspiration. For silica treatments, the average cohort weights were used to deliver 400 mg/kg silica to male mice and 450 mg/kg silica to female mice in 75 μL saline by oropharyngeal aspiration. Silica (MIN-U_SIL5, US Silica) was baked in hydrochloric acid at 110°C overnight, and then washed in sterile saline as previously described (23). Scube2-CreER mice were injected i.p. with 2 mg tamoxifen (MilliporeSigma, T5648) dissolved in olive oil (MilliporeSigma, O1514, 20 mg/mL) once daily for 2 weeks, followed by a 2-week washout period prior to treatment with bleomycin or silica. Cthrc1-CreER mice were injected i.p. with 2 mg tamoxifen dissolved in olive oil once daily for 6 days after bleomycin treatment.

### EdU treatments.

For continuous labeling experiments, EdU (Thermo Fisher Scientific, A10044) was dissolved in distilled water (1 mg/mL) and changed every other day for 21 days. For all pulse-labeling experiments, EdU was dissolved in sterile PBS (10 mg/mL) using a 50°C water bath, and then diluted in sterile PBS to deliver 50 mg/kg mouse body weight EdU in 200 μL PBS. To measure the rolling average of EdU uptake over a 4-day period (Supplemental Figure 1), mice were injected i.p. with EdU once daily for 4 days prior to harvesting the lungs. To measure EdU uptake during the 24 hours prior to harvesting, EdU was injected i.p. once 23 hours prior to harvesting and again at 1 hour prior to harvesting. For PCLSs, EdU was dissolved in DMEM/F12 + GlutaMAX (Thermo Fisher Scientific, 10565018) and added to the culture media at a 20 μM concentration. Lung slices without EdU were used as negative controls for flow cytometric gating. For all experiments, EdU uptake was measured by flow cytometry using the Click-iT Plus EdU Alexa Fluor 647 Kit (Thermo Fisher Scientific, C10634).

### Tissue dissociation.

Mouse lungs were dissociated as previously described (23). In brief, mouse lungs were perfused with PBS through the right ventricle, and then the left lung was minced with scissors. The tissue was suspended in 1 mL protease solution [0.25% collagenase A, MilliporeSigma), 1 U/mL dispase (MilliporeSigma), 2,000 U/mL DNase I (MilliporeSigma) in HBBS (Thermo Fisher Scientific)] and then transferred into a 24-well plate and placed in a 37°C incubator for 60 minutes with trituration by micropipette every 20 minutes. The single-cell suspension was filtered through a 70 μM cell strainer (BD Biosciences), washed with PBS and resuspended in 1 mL ACK RBC lysis buffer (Gibco, Thermo Fisher Scientific) for 90 seconds, and then washed with 10% FBS, and resuspended in 1% BSA (Fisher BioReagents). For dissociation of human lung tissue, 3 PCLSs, (approximately 1 cm height × 1 cm width × 500 μm thick each) were minced with scissors then suspended in 3 mL protease solution, with all other downstream steps being identical to those for the mouse lung dissociation.

### Pulse oximetry.

Mice were gently shaved around the neck using an electric trimmer, and a MouseOx pulse oximetry collar (Starr Life Sciences) was secured to the neck to enable continuous measurement of oxygen saturation. Readings were recorded for a minimum of 5 minutes, and for each animal, the highest 1,500 pulse oximetry values were averaged.

### Flow cytometry.

Following lung tissue dissociation, 3 × 10^6^ cells were used for flow cytometry. Cells were incubated with primary conjugated antibodies in 1% BSA PBS for 30 minutes on ice. CountBright Plus Ready Tubes (Thermo Fisher Scientific, C40000) were used for absolute cell counts. The following anti-mouse reagents were used at 1:100 concentrations unless otherwise specified: LIVE/DEAD stain (DAPI 0.1 μg/mL; Live-or-Dye 330/410 [1:500], Biotium); CD45 (clone 30-F11, BV786, BD Biosciences); CD31 (clone 390, BV605, BD Biosciences); EPCAM (clone G8.8, PE, BV421 BioLegend); MCAM (clone ME-9F1, Alexa Fluor 488, BioLegend); and CD9 (clone MZ3, APC/Fire 750, BioLegend). The following anti-human reagents were used at 1:100 concentrations unless otherwise specified: LIVE/DEAD stain (Live-or-Dye 330/410 [1:200], Biotium; CD45 (clone HI30, APC-Fire 750, Biotin, BioLegend); CD31 (clone WM59, BV605, BV786, Biotin, BD Biosciences); EPCAM (clone 9C4, PE, BioLegend); MCAM (clone P1H12, PE-Cy7, BioLegend); and streptavidin (BV605, BD Biosciences). Data were acquired with the Aria Fusion (BD Biosciences) using BD FACSDiva and analyzed using FlowJo software (BD). Day 21 was selected for FACS quantification of tdTomato^+^ fibroblasts on the basis of our prior work identifying this as the peak of Cthrc1^+^ fibroblast accumulation in bleomycin-challenged lungs (23).

### Real-time qPCR.

Approximately 3,000 cells were sorted directly into 400 μL TRIzol (Thermo Fisher Scientific, 15596026), and RNA was isolated according to the manufacturer’s instructions. RNA was reverse transcribed using SuperScript IV VILO Master Mix with the ezDNase Enzyme kit (Thermo Fisher Scientific, 11756050). Real-time qPCR was performed using PowerUp SYBR Green Master Mix (Thermo Fisher Scientific, A25742) with a Quant Studio 4 (Applied Biosystems). qPCR primers are listed in Supplemental Table 1. For qPCR analysis of Ect2, Scube2-CreER tdTomato^+^ fibroblasts were initially sorted on post-injury day 21, according to the same strategy used for Esco2. However, Ect2 transcripts were undetectable at this time point. The sort was therefore repeated on day 10 following bleomycin injury, a time point that captures the peak of fibroblast proliferation in this model.

### Hydroxyproline assay.

Hydroxyproline was measured as previously described (23). The left lobe was homogenized using a Tissue Tearer (Biospec Products, 985370-395), precipitated with trichloroacetic acid, baked in hydrochloric acid at 110°C overnight, reconstituted in water, and finally analyzed by colorimetric chloramine T assay. Day 28 was selected for hydroxyproline measurement to align with prior studies showing measurable collagen accumulation at this time point in both silica- and bleomycin-induced fibrosis (74, 75).

### Human PCLSs.

Fibrotic lung tissues were obtained at the time of lung transplantation from patients with a diagnosis of IPF or silicosis. Basilar subpleural lung tissue was washed in PBS 3 times for 10 minutes each. Eighteen gauge needles were used to instill 2% agarose (Thermo Fisher Scientific, BP1360-100) in PBS into the large airways, and then left on ice for 30 minutes to solidify. The inflated lung was cut into approximately 2 cm^3^ cubes, mounted onto the stage of a Leica VT1200 vibratome. Sections of 500 μm thickness were cut at 0.70 mm/s, and then cut with scissors into slices (~1 cm height × 1 cm width) and placed into a 24-well plate in PBS on ice. Lung slices were then transferred into new 24-well plates containing DMEM/F12 + GlutaMAX (Thermo Fisher Scientific, 10565018) supplemented with Fungizone (Thermo Fisher Scientific, 15290026, 1:400), Primocin (InvivoGen, ant-pm-1, 100 μg/mL), insulin-transferrin-selenium (Thermo Fisher Scientific, 41-400-045, 1:100), EdU (Thermo Fisher Scientific, A10044, 20 μM), and 1% FBS. Media were changed daily. Lung slices were cultured for 2 days at 37°C, 8% CO_2_ and then washed in PBS and diced using scissors. Three lung slices were pooled into a single technical replicate. Three technical replicates (*n* = 9 lung slices total) per donor were analyzed by flow cytometry and then averaged to obtain a single value for plotting.

### Histology.

The right lung was inflated with 4% PFA in PBS under constant pressure of 25 cm H_2_O, tied off, and then fixed in 4% PFA in PBS overnight at 4°C. Mouse lungs were then washed in PBS for 1 hour. The right upper lobe was used for cleared whole lung imaging. For confocal imaging, the right lung or human PCLSs were transferred into 30% sucrose in PBS overnight and then embedded in Tissue-Plus Optimal Cutting Temperature compound (Thermo Fisher Scientific, 23-730-571) for cryosectioning.

### Confocal imaging of human PCLSs.

Cryosections (12 μm thick) of human PCLSs were mounted on Superfrost Plus slides (Thermo Fisher Scientific), rinsed in PBS, and photobleached to reduce autofluorescence using 3 rounds of quenching solution as previously described (76), with 45W LED panels (Amazon, B0DWMZ4K2M). EdU incorporation was detected using the Alexa Fluor 488 EdU Kit (Thermo Fisher Scientific, C10337) and counterstained with DAPI. ISH for Cthrc1 was performed using the RNAscope Multiplex Fluorescent Reagent Kit version 2 (ACD). Images were acquired on a Stellaris confocal microscope (Leica).

### Confocal imaging of fibrotic and control mouse lungs.

Cryosections (12 μm thick) of mouse lung were mounted on Superfrost Plus glass slides (Thermo Fisher Scientific), washed in PBS, and counterstained with DAPI. Representative images of DAPI and tdTomato^+^ cells were acquired on a Stellaris confocal microscope (Leica).

### Confocal imaging of Brainbow2.1 Confetti mouse lungs.

Cryosections (100 μm thick) of mouse lung were washed in PBS and then transferred into CUBIC-L (TCI, T3740) and incubated at 37°C on an orbital shaker (235*g*) for 24 hours. Lung sections were stained with DRAQ7 (BioLegend) in PBS for 1 hour at room temperature, transferred to a 24-well glass bottom plate (Cellvis, P24-1.5H-N), and then immersed in CUBIC R+M (TCI, T3741) for 2 hours at room temperature. *Z*-stack optical sections (50 μm) were obtained using an inverted Leica Stellaris Confocal Microscope. DAPI, GFP, YFP, and RFP channels were acquired for Scube2-CreER-Brainbow2.1 mouse lung sections. Cthrc1-CreER labels a small percentage of lineage-positive cells at baseline (23), so we only quantified cytoplasmic YFP^+^ and cytoplasmic RFP^+^ clones with clear fibroblast morphology for Cthrc1-CreER Brainbow2.1 mice. 3D maximum projection images were generated in Imaris Viewer 10.2.0.

### Picrosirius red imaging.

Six cryosections (12 μm) were collected per lung, spaced at 30 μm intervals to ensure even sampling across the tissue depth. Sections were mounted on Superfrost Plus slides (Thermo Fisher Scientific), rinsed in PBS, and stained with Picrosirius red to visualize fibrillar collagen. For each biological replicate, 3 random fields were acquired from saline-treated lungs, and 6 lesion-enriched fields were imaged from fibrotic lungs. Images were obtained using a ×20 objective on a DMi8 inverted wide-field microscope (Leica). Collagen content was quantified using the ImageJ color deconvolution macro (77) and expressed as the percentage of area positive for Picrosirius red. We selected day 28 for Picrosirius red imaging to align with prior studies showing measurable collagen accumulation at this time point in both silica- and bleomycin-induced fibrosis (74, 75).

### Cleared whole lung and light sheet imaging.

After fixation in 4% PFA, the right upper lobe was transferred into CUBIC-L (TCI, T3740) and incubated at 37°C on an orbital shaker (180 rpm) for 24 hours 3 times, and then washed in PBS as previously described (23). The cleared right upper lobe was RI matched in CUBIC R+M (TCI, T3741) for 36 hours at room temperature, and then transferred into RI 1.520 Mounting Solution for Cubic R+ (TCI, M3294). Images were acquired on the Nikon AZ100 Light Sheet microscope using the RFP channel to detect tdTomato and GFP channel to detect autofluorescence. 3D maximum projection images were generated using Imaris Viewer 10.2.0.

### scRNA-Seq analysis for publicly available mouse lung dataset.

We used our previously published scRNA-Seq dataset of bleomycin-treated Scube2-CreER; Ai14 mouse lung fibroblasts (GSE210341) (23) uploaded to the GEO database. Using Seurat version 5, the proliferating fibroblast cluster was divided into subsets and reclustered using NormalizeData, FindVariableFeatures, RunFastMNN, RunUMAP (1:20 dimensions), FindNeighbors (1:20 dimensions), and FindClusters (0.3 resolution). Differentially expressed genes for each of the proliferating fibroblast clusters were identified using the FindMarkers function (minimum percent, 0.25; log fold change [FC] threshold, 0.25). tdTomato^+^ cells were defined by a natural log–normalized tdTomato expression level of greater than 3.5 to quantify the percentage of tdTomato^+^ cells per cluster as previously described (23). Scaled average gene expression was used to calculate the Spearman correlation coefficients for each cell cluster and plotted using pheatmap. Ggplot was used to generate scatter plots of differentially expressed genes comparing proliferating fibrotic fibroblasts (day 7) versus reference alveolar fibroblasts (all days) and nonproliferating fibrotic fibroblasts (day 7) versus reference alveolar fibroblasts (all days). Day 7 was selected because this time point had the highest frequency of proliferating fibroblasts.

### scRNA-Seq analysis for the publicly available human lung dataset.

We used our previously published (23) combined scRNA-Seq Seurat object of pathologic and alveolar fibroblasts derived from normal and fibrotic human lungs by Tsukui (GSE132771) (23), Adams (GSE147066) (78), and Haberman (GSE135893) (63) uploaded to the GEO database. We excluded cells from the Adams (GSE147066) dataset because Adams et al. regressed out cell-cycle genes. Cell-cycle scores were then calculated using the cell-cycle scoring package in Seurat version 5. We observed a lower frequency of high cell-cycle scores in cells derived from the Tsukui (GSE132771) dataset, which may be due to longer storage of fresh tissue prior to tissue dissociation. Thus, we focused exclusively on cells derived from the Habermann dataset (GSE135893). To define proliferating versus nonproliferating cells, we used the density-based spatial clustering of applications with noise (DBSCAN) algorithm to identify hierarchical clusters based on cell-cycle scores (eps 0.037, minPts 3). The decision tree model (rpart package) was then used to identify optimal cutoffs of cell-cycle scores to delineate proliferating from nonproliferating cells (Supplemental Figure 4).

### Statistics.

Unpaired parametric, 2-tailed *t* tests were used for single comparisons between 2 groups. Two-way ANOVA with Tukey’s correction was used for multiple comparisons between 3 or more groups. A *P* value less than 0.05 was considered significant. Data are presented as the mean ± SEM.

### Study approval.

Animal studies were approved by the IACUC of UCSF, San Francisco, California. The protocol for harvesting human fibrotic lung tissue was approved by the UCSF Committee on Human Research, and written informed consent was received prior to participation. The study was conducted according to Declaration of Helsinki principles.

### Data availability.

The scRNA-Seq analysis was performed on publicly available data from the Gene Expression Omnibus (GEO) database (GSE132771 and GSE135893). The Supporting Data Values file is included in the supplemental materials.

## Author contributions

CM and DS conceived the studies, interpreted the data, and wrote the manuscript. CM performed all the experiments. TT contributed Scube2-CreER and Cthrc1-CreER mice and provided feedback on experimental design, methods, and manuscript revisions. IK provided feedback on experimental design and data interpretation. XR and WQ assisted with harvesting mouse lungs for the hydroxyproline experiments. PW and MM procured human lung samples.

## Funding support

This work is the result of NIH funding, in whole or in part, and is subject to the NIH Public Access Policy. Through acceptance of this federal funding, the NIH has been given a right to make the work publicly available in PubMed Central.

- National Heart, Lung, and Blood Institute (NHLBI), NIH grant 5R01HL142568-04.

## Supplementary Material

Supplemental data

Supporting data values

## Version 1 08/28/2025

In-Press Preview

## Version 2 11/17/2025

Electronic publication

## Version 3 09/14/2026

Figure 4B has been updated
