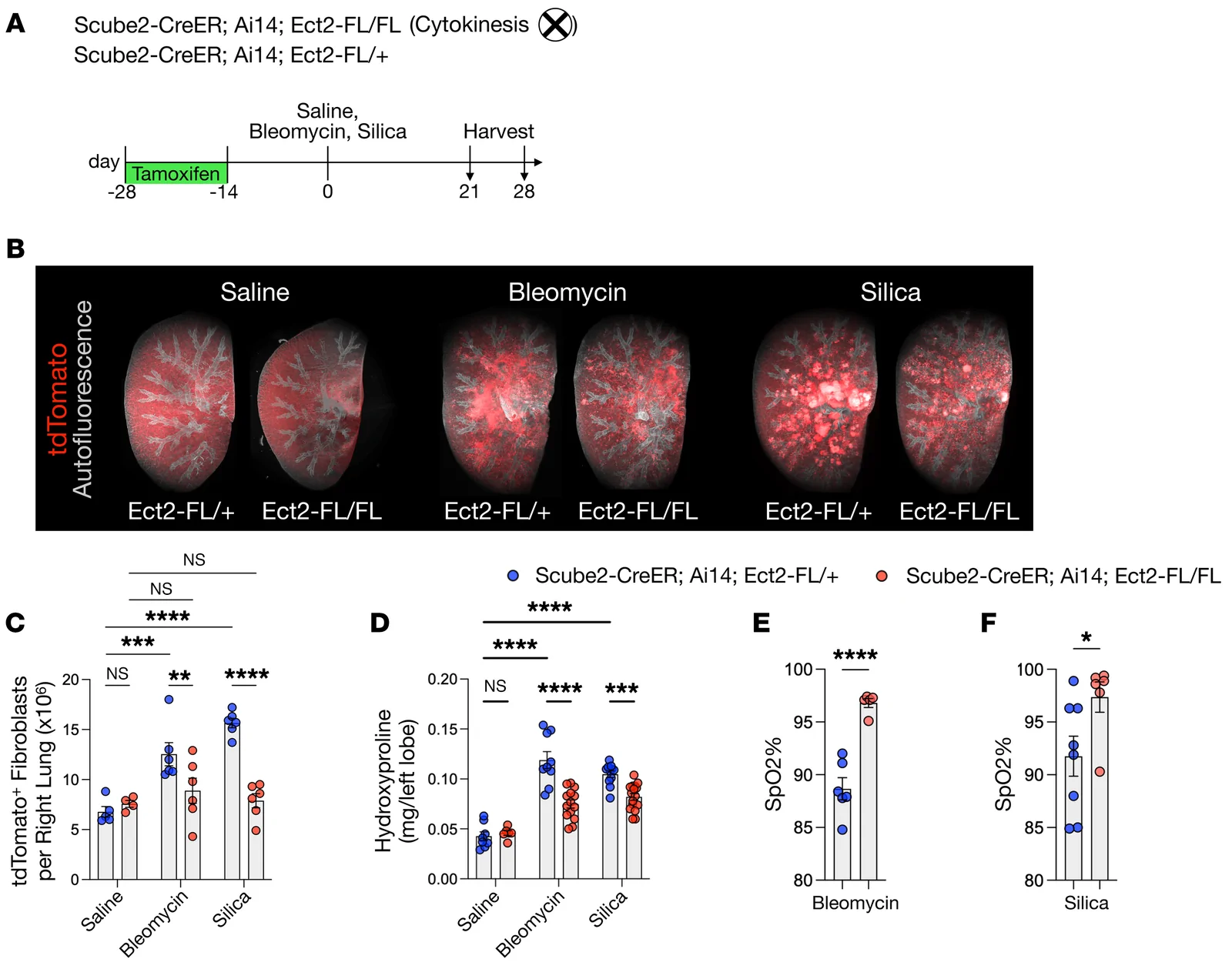# Erratum to Clonal expansion of alveolar fibroblast progeny drives pulmonary fibrosis in mouse models

**DOI:** 10.1172/JCI212784

**Published:** 2026-09-15

**Authors:** Christopher Molina, Tatsuya Tsukui, Imran S. Khan, Xin Ren, Wenli Qiu, Michael Matthay, Paul Wolters, Dean Sheppard

Original citation: *J Clin Invest*. 2025;135(22):e191826. https://doi.org/10.1172/JCI191826

Citation for this erratum: *J Clin Invest*. 2026;136(18):e212784. https://doi.org/10.1172/JCI212784

During preparation of this manuscript, *JCI* staff inadvertently introduced an error in Figure 4B, which was duplicated from Figure 3B. The correct figure is shown below, and the HTML and PDF versions of the article have been updated online.

The *JCI* regrets the error.

## Figures and Tables

**Figure 4:**