# Supplementary material for: Clonal expansion of alveolar fibroblast progeny drives pulmonary fibrosis in mouse models
Source: J Clin Invest. 2025 Aug 28;135(22):e191826. doi: 10.1172/JCI191826 (PMC12685133; doi:10.1172/JCI191826)
Supplement: Supplemental data [file jci-135-191826-s006.pdf]

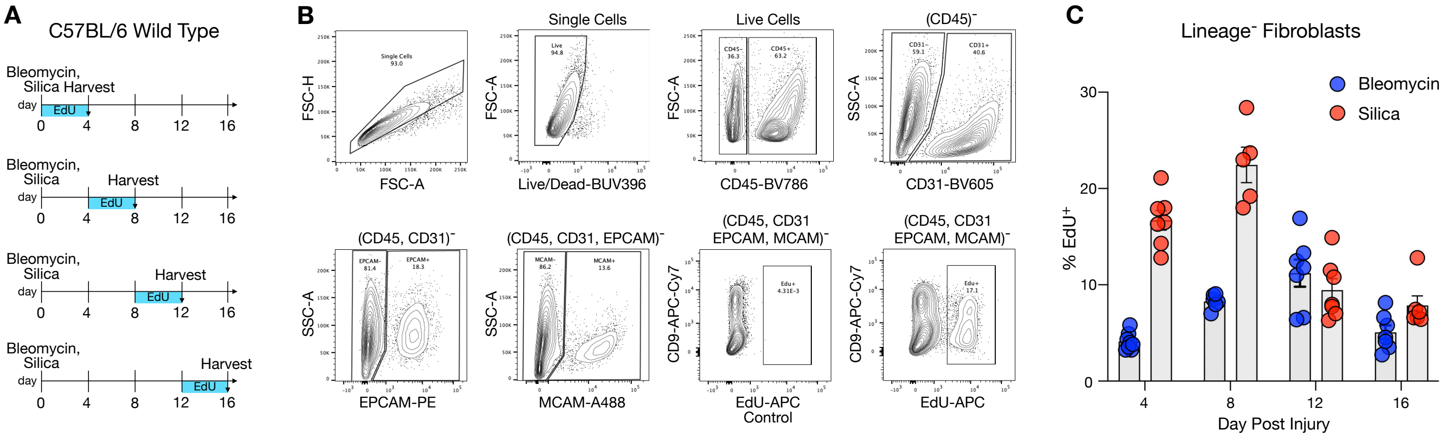

Supplemental Figure 1: Proliferation Kinetics of Lineage- Fibroblasts in Fibrotic Lungs. (**A**) C57BL/6 wild type mice were challenged with bleomycin or silica to induce pulmonary fibrosis then treated with daily injections of EdU for 4 days prior to harvest. Mice were harvested on days 4, 8, 12, and 16 post-injury and analyzed by flow cytometry. (**B**) Flow gating strategy for quantifying EdU uptake in lineage (CD45, CD31, EPCAM, MCAM)-negative lung fibroblasts. EdU-untreated mice were used as the EdU gating control. (**C**) Four-day average of % EdU uptake in lineage- fibroblasts after fibrotic lung injury with bleomycin or silica.

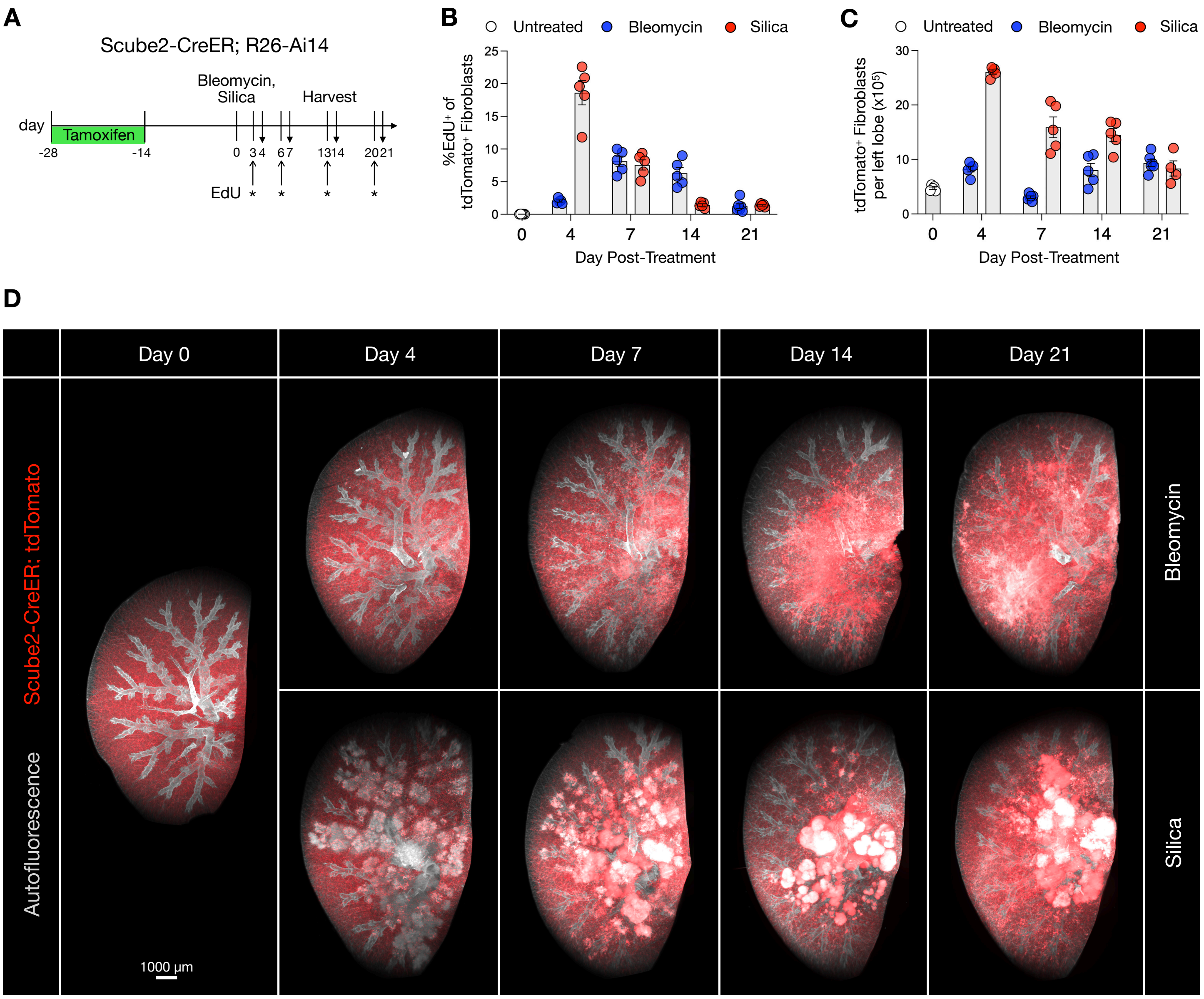

Supplemental Figure 2: Proliferation Kinetics of Alveolar Fibroblast Descendants in Two In Vivo Models of Pulmonary Fibrosis. (**A**) Scube2-CreER/Rosa26-Ai14 mice were injected with tamoxifen to label alveolar fibroblasts with tdTomato, challenged with bleomycin or silica, then treated with EdU (*) 24 hours prior to harvest on days 4, 7, 14, and 21 post-injury. Total numbers of (CD45, CD31, MCAM, EPCAM)-negative tdTomato+ fibroblasts and their percentage of EdU uptake were quantified by flow cytometry. (**B**) Percentage of EdU uptake in tdTomato+ fibroblasts in bleomycin and silica-treated lungs, showing peak EdU uptake in silica-treated lungs at day 4 post-injury, and peak EdU uptake in bleomycin-treated lungs on day 7-post injury. (**C**) Quantification of absolute numbers of tdTomato+ fibroblasts post-injury. (**D**) 3D maximum projection images of cleared whole lungs showing the temporal emergence of tdTomato+ cell aggregates in bleomycin and silica-treated fibrotic lungs.

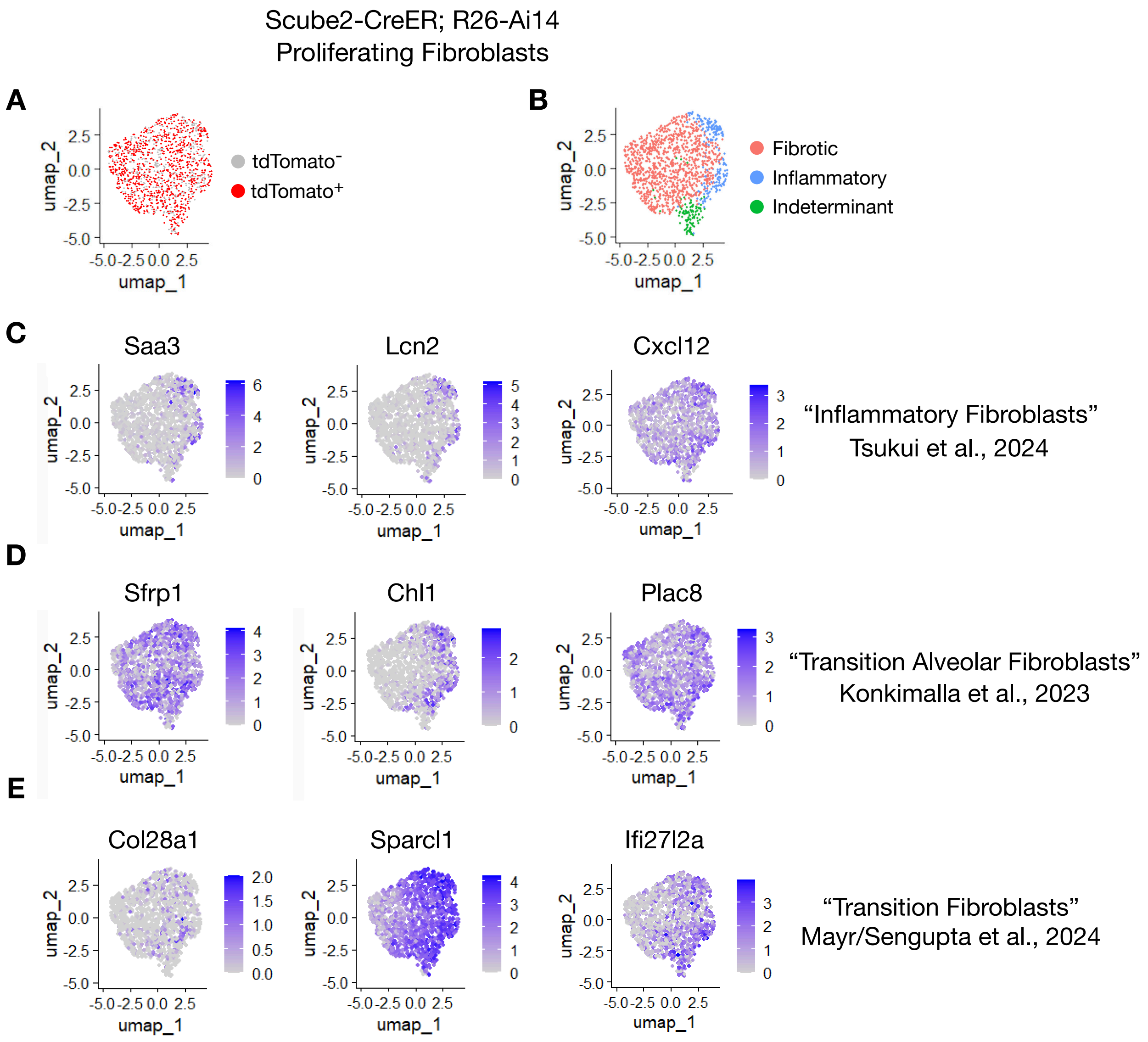

Supplemental Figure 3: Fibroblast Subpopulation Comparison to Previous Studies. (**A-B**) UMAP plot of proliferating fibroblasts from Figure 2, showing all subpopulations of proliferating fibroblasts were predominantly labeled by tdTomato. (**C-D**) Expression of genes related to previously described fibroblast subpopulations on UMAP feature plots of proliferating fibroblasts.

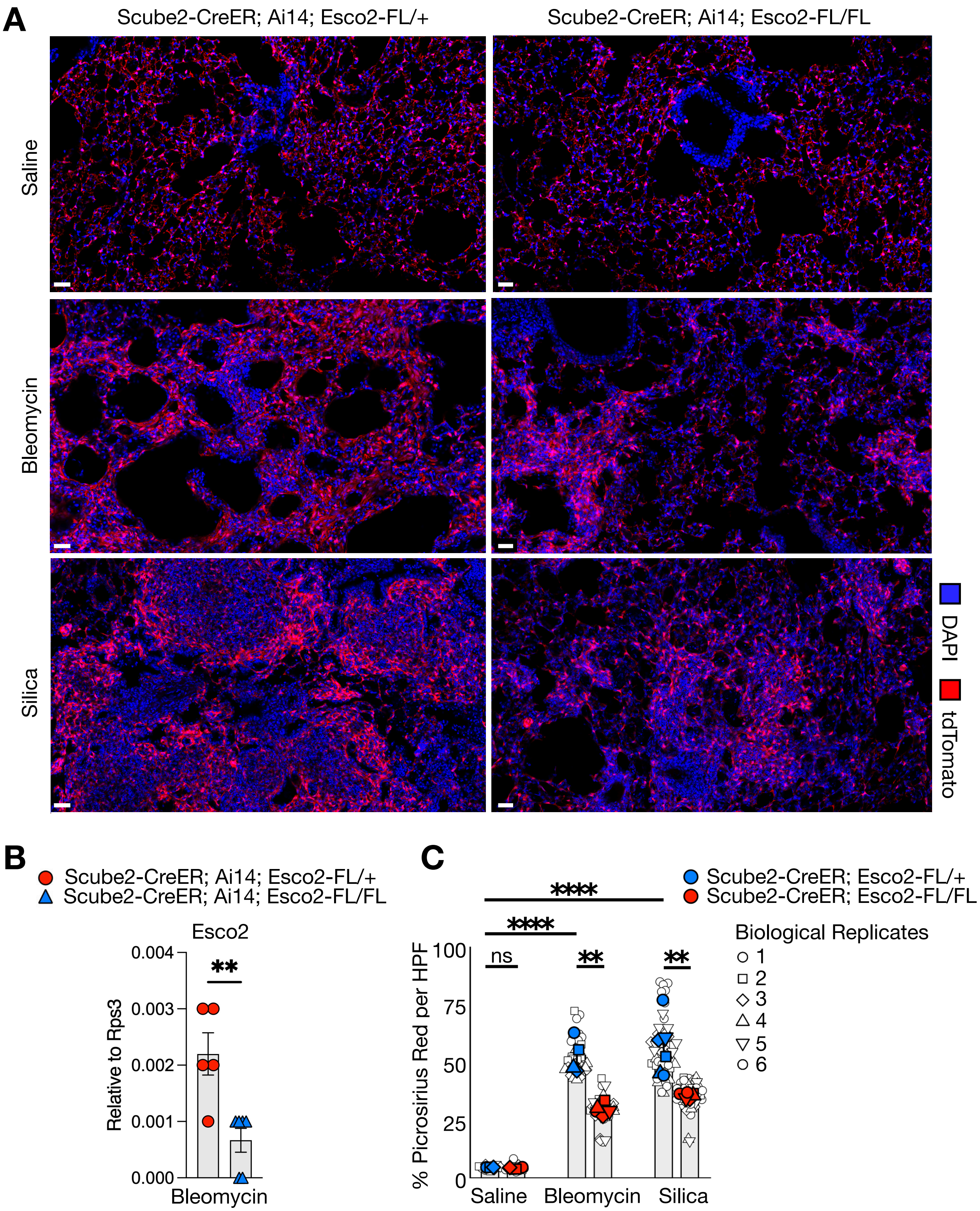

Supplemental Figure 4: Fibroblast depletion and collagen reduction in Scube2-CreER; Ai14; Esco2-deleted mice. **(A)** Confocal images of lungs harvested at day 21 post-treatment show reduced density and size of tdTomato⁺ fibroblast aggregates in bleomycin- and silica-treated Esco2-FL/FL mice compared to FL/+ controls. Scale bar = 50 µm. **(B)** qPCR from FACS-sorted tdTomato⁺ fibroblasts at day 21 post-bleomycin shows a 70% reduction in Esco2 expression in Esco2-FL/FL mice. **(C)** Picrosirius red staining of lungs at day 28 post-treatment shows a 44% reduction in collagen content per high-power field in bleomycin-treated and 38% reduction in silica-treated Esco2-FL/FL mice compared to controls. 2-way ANOVA with Tukey's correction for multiple comparisons. Error bars denote SEM. **$p<0.01$, ****$p<0.0001$.

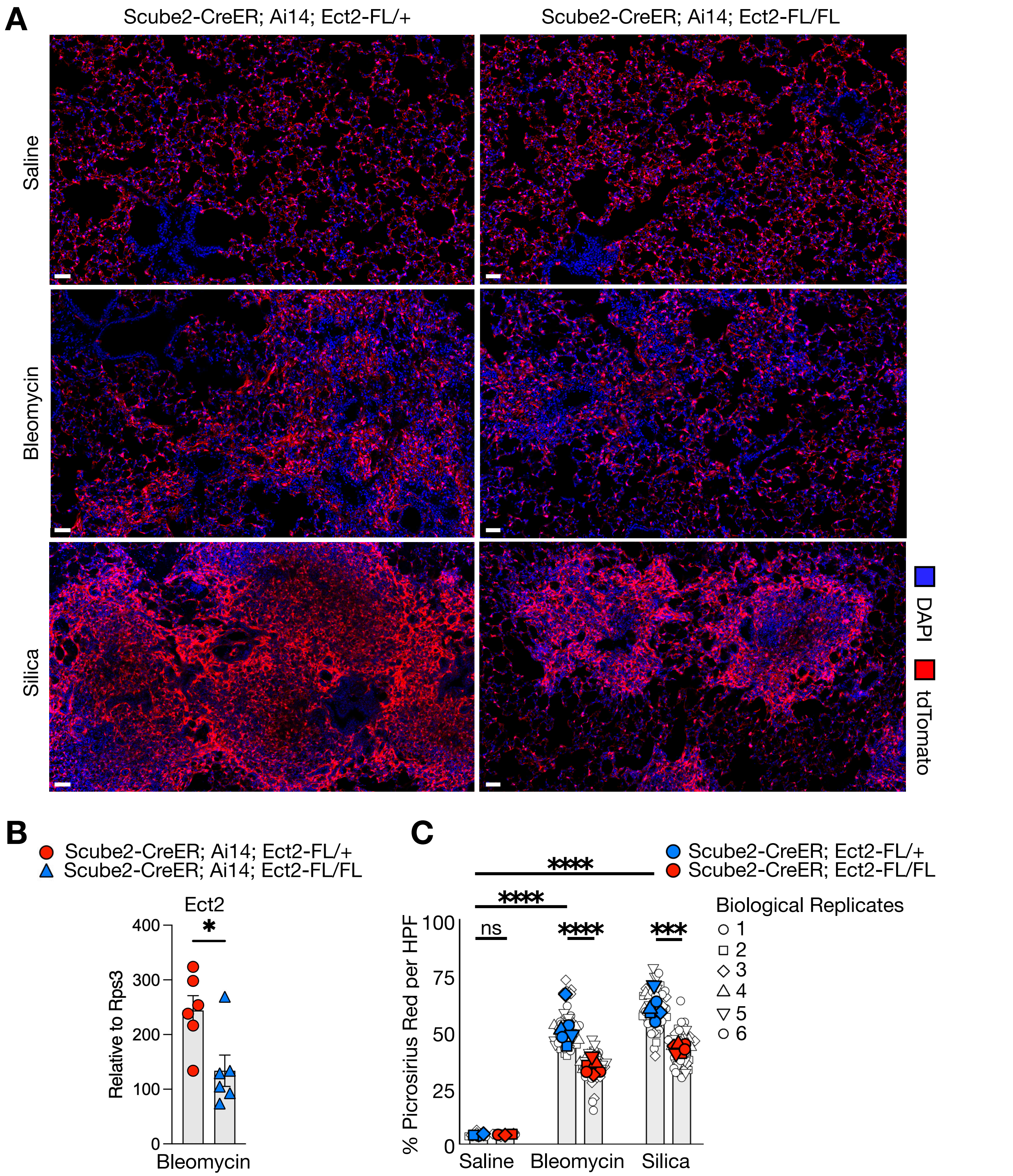

Supplemental Figure 5: Inhibition of fibroblast proliferation and collagen reduction in Scube2-CreER; Ai14; Ect2-deleted. mice. (**A**) Confocal images of lungs harvested at day 21 post-treatment show reduced size and organization of tdTomato⁺ fibroblast aggregates in bleomycin- and silica-treated Ect2-FL/FL mice compared to FL/+ controls. Scale bar = 50 µm. (**B**) qPCR from FACS-sorted tdTomato⁺ fibroblasts at day 10 post-bleomycin shows a 45% reduction in Ect2 expression in Ect2-FL/FL mice. (**C**) Picrosirius red staining of lungs at day 28 post-treatment shows a 34% reduction in collagen content per high-power field in bleomycin-treated and 24% reduction in silica-treated Ect2-FL/FL mice compared to controls. 2-way ANOVA with Tukey's correction for multiple comparisons. Error bars denote SEM. ***$p<0.001$ ****$p<0.0001$.

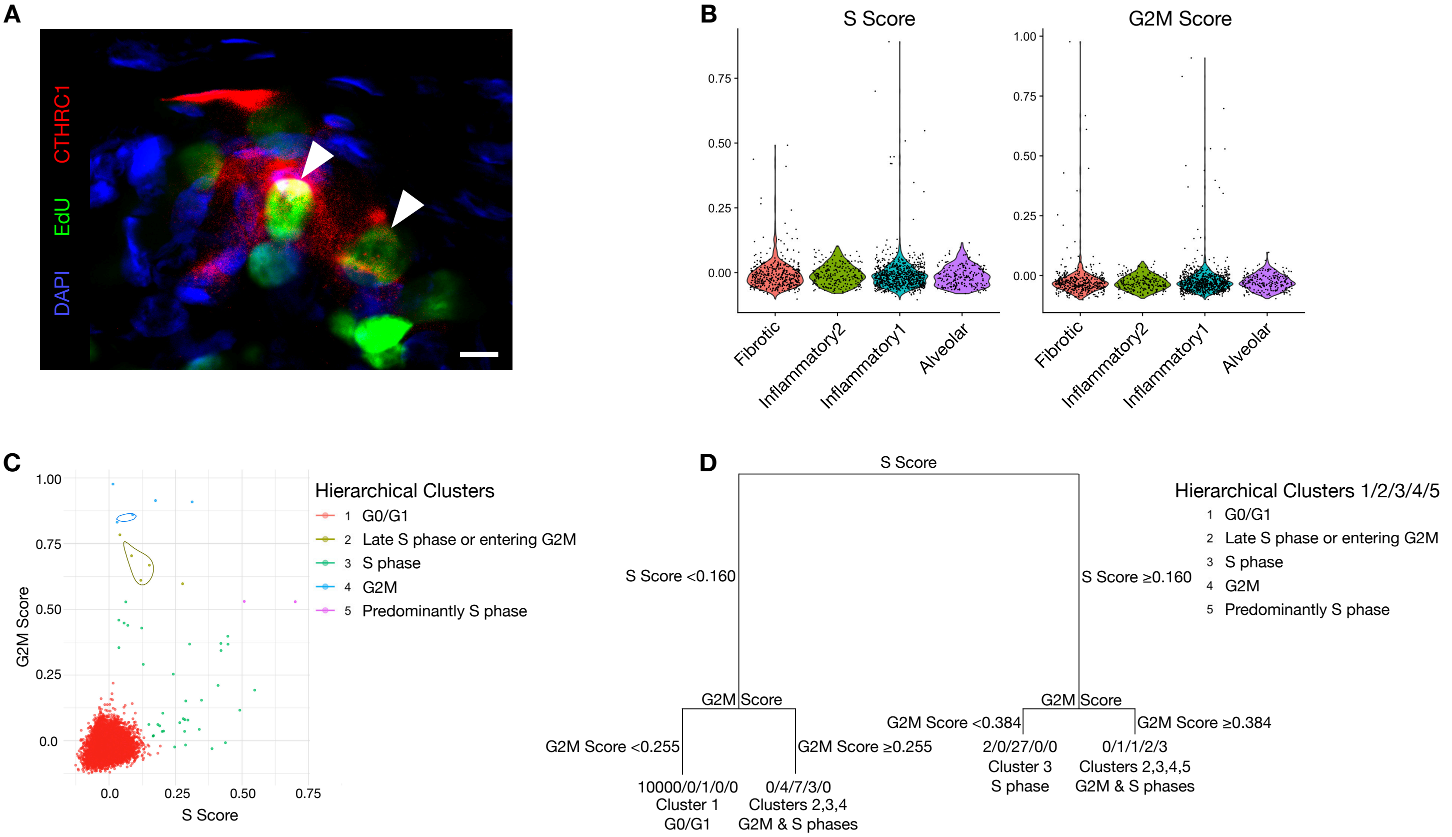

Supplemental Figure 6: Identification of proliferating fibroblast subtypes in human precision-cut lung slices and scRNA-seq analysis of fibrotic lungs. (**A**) RNAscope imaging of IPF precision-cut lung slices (PCLS) from Figure 5b shows Cthrc1$^+$ EdU$^+$ cells (arrowheads). Scale bar = 10 µm. (**B**) Cell cycle scoring was applied to fibroblast subsets from the reanalyzed Habermann et al. IPF single-cell atlas by Tsukui et al. 2024. (**C**) Scatterplot of S-phase and G2/M-phase scores for each fibroblast, clustered using DBSCAN (Density-Based Spatial Clustering of Applications with Noise). (**D**) A machine learning decision tree model identified optimal thresholds for S-phase (≥0.201) and G2/M-phase (≥0.1859) scores to classify cells as proliferative (DBSCAN Clusters 2, 3, 4, 5) or non-proliferative (Cluster 1). This classification accurately defined 99.99% of DBSCAN Cluster 1 (G0/G1) cells as non-proliferative. Cell numbers per DBSCAN cluster are reported as $n_1/n_2/n_3/n_4/n_5$ corresponding to Clusters 1 through 5.

Supplementary Table 1. qPCR Primers

Rps3 F	cggtgcagatttccaagaag
Rps3 R	ggacttcaactccagagtagcc
Scube2 F	CCTCTCTCAGAAGCAAACAGC
Scube2 R	GTCCTGACGGTGACGACAT
Pdgfra F	cggagcctgagctttgag
Pdgfra R	gccctgtgaggagacagc
Tcf21 F	CATTCACCCAGTCAACCTGA
Tcf21 R	CCACTTCCTTCAGGTCATTCTC
Npnt F	cagtgccaacctttctacgtc
Npnt R	tgtttgcactgtggttgaca
Cthrc1 F	aagcaaaaagcgctgatcc
Cthrc1 R	cctgctggtccttgtagacac
Col1a1 F	AGACATGTTCAGCTTTGTGGAC
Col1a1 R	GCAGCTGACTTCAGGGATG
Postn F	aagctgcggcaagacaag
Postn R	tcaaatctgcagcttcaagg
Tnc F	gggctatagaacaccgatgc
Tnc R	catttaagtttccaatttcaggttc
Esco2 F	GACCTATAAGCCAGTTGTGGAC
Esco2 R	TCCGCCTTGGAGTGTAACTTG
Ect2 F	ACCAGTTGGGGATGAAAGGTG
Ect2 R	GCACGAGCGTCCATCTGAA

Supplementary Table 2: Donor characteristics for human precision-cut lung slice experiments.

Non-diseased Human Lung Donors

Diagnosis	Age	Sex	Ethnicity	Smoking Status
Natural Causes	64	Male	Non-hispanic white	Unknown
Blunt Head Trauma	50	Female	Non-hispanic white	Former smoker
Hemorrhagic Stroke	65	Male	Non-hispanic white	Never smoker
Natural Causes	63	Male	Hispanic	Never smoker
Average Age	61			

Fibrotic Human Lung Donors

Diagnosis	Age	Sex	Ethnicity	Smoking Status
Idiopathic Pulmonary Fibrosis	67	Female	Non-hispanic white	Never smoker
Familial Idiopathic Pulmonary Fibrosis	55	Male	American Indian	Former smoker
Idiopathic Pulmonary Fibrosis	62	Female	Non-hispanic white	Never smoker
Idiopathic Pulmonary Fibrosis	67	Male	Non-hispanic white	Former smoker
Idiopathic Pulmonary Fibrosis	56	Male	Hispanic	Former smoker
Idiopathic Pulmonary Fibrosis	69	Male	Non-hispanic white	Former smoker
Idiopathic Pulmonary Fibrosis	61	Male	Non-hispanic white	Former smoker
Idiopathic Pulmonary Fibrosis	63	Male	White	Never smoker
Pulmonary Silicosis	41	Male	Hispanic	Never smoker
Average Age	60			
